# Unraveling the Secrets of a Double-Life Fungus by Genomics: *Ophiocordyceps australis* CCMB661 Displays Molecular Machinery for Both Parasitic and Endophytic Lifestyles

**DOI:** 10.3390/jof9010110

**Published:** 2023-01-13

**Authors:** Thaís Almeida de Menezes, Flávia Figueira Aburjaile, Gabriel Quintanilha-Peixoto, Luiz Marcelo Ribeiro Tomé, Paula Luize Camargos Fonseca, Thairine Mendes-Pereira, Daniel Silva Araújo, Tarcisio Silva Melo, Rodrigo Bentes Kato, Jacques Hubert Charles Delabie, Sérvio Pontes Ribeiro, Bertram Brenig, Vasco Azevedo, Elisandro Ricardo Drechsler-Santos, Bruno Silva Andrade, Aristóteles Góes-Neto

**Affiliations:** 1 Department of Biological Sciences, Universidade Estadual de Feira de Santana, Av. Transnordestina, s/n, Novo Horizonte, Feira de Santana 44036-900, BA, Brazil; 2 Laboratory of Integrative Bioinformatics, Preventive Veterinary Medicine Department, Veterinary School, Universidade Federal de Minas Gerais, Belo Horizonte 31270-901, MG, Brazil; 3 Laboratory of Molecular and Computational Biology of Fungi, Institute of Biological Sciences, Universidade Federal de Minas Gerais, Av. Antônio Carlos, 6627, Pampulha, Belo Horizonte 31270-901, MG, Brazil; 4 Program in Bioinformatics, Loyola University Chicago, Chicago, IL 60660, USA; 5 Laboratory of Myrmecology, Centro de Pesquisa do Cacau, Ilhéus 45600-000, BA, Brazil; 6 Department of Agricultural and Environmental Sciences, Universidade Estadual de Santa Cruz, Ilhéus 45600-970, BA, Brazil; 7 Laboratory of Ecology of Diseases and Forests, Nucleus of Biological Science, Campus Morro do Cruzeiro, Universidade Federal de Ouro Preto, Ouro Preto 35402-163, MG, Brazil; 8 Institute of Veterinary Medicine, Burckhardtweg, University of Göttingen, 37073 Göttingen, Germany; 9 Laboratory of Cellular and Molecular Genetics, Universidade Federal de Minas Gerais, Belo Horizonte 31270-901, MG, Brazil; 10 Biological Sciences Center, Universidade Federal de Santa Catarina, Florianópolis 88040-900, SC, Brazil; 11 Department of Biological Sciences, Universidade Federal do Sudoeste da Bahia, Av. José Moreira Sobrinho, s/n, Jequiezinho, Jequié 45205-490, BA, Brazil

**Keywords:** Ophiocordycipitaceae, entomopathogenicity, fungal genomics, insect-fungus symbiosis, zombie-ant fungus

## Abstract

*Ophiocordyceps australis* (Ascomycota, Hypocreales, Ophiocordycipitaceae) is a classic entomopathogenic fungus that parasitizes ants (Hymenoptera, Ponerinae, Ponerini). Nonetheless, according to our results, this fungal species also exhibits a complete set of genes coding for plant cell wall degrading Carbohydrate-Active enZymes (CAZymes), enabling a full endophytic stage and, consequently, its dual ability to both parasitize insects and live inside plant tissue. The main objective of our study was the sequencing and full characterization of the genome of the fungal strain of *O. australis* (CCMB661) and its predicted secretome. The assembled genome had a total length of 30.31 Mb, N50 of 92.624 bp, GC content of 46.36%, and 8,043 protein-coding genes, 175 of which encoded CAZymes. In addition, the primary genes encoding proteins and critical enzymes during the infection process and those responsible for the host–pathogen interaction have been identified, including proteases (Pr1, Pr4), aminopeptidases, chitinases (Cht2), adhesins, lectins, lipases, and behavioral manipulators, such as enterotoxins, Protein Tyrosine Phosphatases (PTPs), and Glycoside Hydrolases (GHs). Our findings indicate that the presence of genes coding for Mad2 and GHs in *O. australis* may facilitate the infection process in plants, suggesting interkingdom colonization. Furthermore, our study elucidated the pathogenicity mechanisms for this *Ophiocordyceps* species, which still is scarcely studied.

## 1. Introduction

Entomopathogenic fungi cause infections in insects and other terrestrial arthropods, such as mites, spiders, and ticks, leading to an observable disease. Most entomopathogenic species are from the hyperdiverse order Hypocreales (Ascomycota), including the families Ophiocordycipitaceae, Cordycipitaceae, and Clavicipitaceae, with different levels of host specificity [1,2,3,4]. Moreover, some of these fungi generally play more than one ecological role in nature as endophytes, antagonists of plant pathogens, possible growth promoters in plants, and establish beneficial relationships with the rhizosphere [5,6,7]. In this sense, many fungi traditionally known as insect pathogens have been isolated as endophytes, especially *Acremonium*, *Beauveria*, *Cladosporium*, *Clonostachys*, *Isaria*, and *Metarhizium*, among others [6,7]. The dual ability of fungi to parasitize insects and plants in Hypocreales (Sordariomycetes) is mainly due to enzymes that penetrate the plant cell wall and insect exoskeleton [8].

Infection by entomopathogenic fungi mainly occurs through infectious spores spread in the environment by wind, which, after recognizing and adhering to the host surface, germinate, differentiate, and penetrate directly into the insect cuticle. Following this, the fungus proliferates and colonizes the host body. In order to accomplish these steps, the fungus produces several enzymes and compounds of secondary metabolism and electrostatic and hydrophobic mechanisms in the initial stages of the infection. These compounds interact with the host immune system to guarantee the success of the infection [9,10,11]. Amongst the main enzymes involved in this process, we can highlight proteinases, lipases, and chitinases [12]. In plant pathogenic fungi, CAZymes, mainly pectinases, cellulases, and hemicellulases, are essential for plant cell wall degradation and, consequently, during host-pathogen interactions [13].

Ophiocordycipitaceae is a diverse family that comprises fungi of ecological, economic, medicinal, and cultural importance. This family was recognized due to the polyphyletism of the genus *Cordyceps* and the wide diversity of strains of that are pathogens of arthropods. Most of the species included in this family have stroma with dark pigmentation and malleability [14]. *Ophiocordyceps* is the family’s most studied and diverse genus, with more than 200 species. Species of this genus have septate ascospores (which do not disintegrate into part-spores) and, usually, cylindrical asci with immersed or superficial perithecium. It is a heterogeneous taxon comprising mainly insect pathogens. The stroma of *Ophiocordyceps* species remains attached to the corpses of the host, and the parasitized insects can be found in exposed environments such as, in the litter, underneath leaves, in stems, or even buried in the soil [14,15,16,17,18].

Among the entomopathogenic fungi, *Ophiocordyceps australis* (Ascomycota, Hypocreales, Ophiocordycipitaceae) is a highly virulent fungus that parasites ants (Hymenoptera), mainly workers, especially from the genera *Neoponera*, *Pachycondyla*, *Paltothyreus*, *Crematogaster*, *Paraponera* and *Odontomachus*, and has a wide geographic distribution in the world. This species is considered a facultative animal parasite, a hemibiotrophic fungus that switches from a biotrophic phase (when the fungus parasitizes the host) in the hemocoel to a saprotrophic phase, developing even after host death [19,20].

*Ophiocordyceps australis* has been studied from an evolutionary genomics perspective, aiming to better understand these host-parasite interactions [12,21]. *Ophiocordyceps* species complex parasitizes ants and secretes bioactive compounds that manipulate the host behavior, including enterotoxins, which are proteins related to pathogenicity [21]. Komboo et al. [12], analyzing genes that code for enterotoxins, pointed out that these genes evolve under positive selection. Furthermore, in the same study, the authors suggested that enterotoxins may be important effectors in host adaptation and co-evolution. Additionally, only fungi that cause infections in plants and insects have genes that code for bacterial toxins [22], and other studies have indeed shown that *Ophiocordyceps sinensis* can infect or colonize plant tissue and endophytically live in plant leaves and roots [8,23]. Therefore, our study aimed to investigate the genome of *O. australis* and its predicted secretome, highlighting the primary genes that contribute to the pathogenicity processes, both in ants and in plants, aiming at a better understanding of the host-parasite interaction as well as the possibility of interkingdom colonization for this species. 

## 2. Materials and Methods

### 2.1. Collection, Isolation, and Maintenance of Fungal Samples

We collected ten ants parasitized by fungal species of *Ophiocordyceps*. Field expeditions for specimen acquisition were carried out in Atlantic Forest ecosystems in Florianópolis, Santa Catarina, Brazil, specifically in the following localities: Costão do Santinho, Unidade de Conservação Ambiental Desterro (UCAD), Morro da Lagoa da Conceição, Naufragados, and Santo Amaro da Imperatriz, from 6 to 13 January 2016. The sample used for fungal identification and genome sequencing in this study, *O. australis* (CCMB661), was isolated from the ant *Neoponera curvinodis* Forel in the Laboratory of Mycology (MIND.Funga), Universidade Federal de Santa Catarina, Florianópolis, Brazil. For isolation, surface disinfection of the macrofungus was performed by successive immersions in 70% alcohol and 1% NaClO, followed by washes in distilled water [24]. Fungal stromata were detached from the hosts, and the incisions were cultivated in potato dextrose agar (PDA), and incubated at 25 °C in the absence of light. The preservation of this strain was performed by adding five mycelium plugs (0.5 cm ø) from the primary matrix to glass bottles containing sterile distilled water and cultivated in Sabouraud dextrose agar (SDA) incubated at 27 °C in the absence of light in the Microbiology Research Laboratory, Universidade Estadual de Feira de Santana, Feira de Santana, Bahia, Brazil.

### 2.2. DNA Extraction, Library Preparation, and Genome Sequencing

For DNA extraction, *O. australis* (CCMB661) stromal mycelium was collected from the plate and transferred to a microtube (2 mL) containing lysis solution buffer for cell extraction using the FastDNA kit (MP Biomedicals, Irvine, CA, USA), in the Laboratory of Molecular and Computational Biology of Fungi (LBMCF), Universidade Federal de Minas Gerais, Belo Horizonte, Brazil. Sample quality was evaluated using agarose gel 1% electrophoresis, and the concentration of DNA was measured in Nanodrop 1000 ND spectrophotometer (Thermo Scientific, Waltham, MA, USA). The sequencing library was prepared from genomic DNA [1 µg] using the NEBNext Fast DNA Fragmentation and Library Preparation Kit (New England Biolabs, Ipswich, NE, USA). Finally, HiSeq 2500 (Illumina, San Diego, CA, USA) was used for the whole genome sequencing.

### 2.3. Quality Control, Assembly, and Structural Annotation of the O. australis (CCMB661) Genome

The quality of the raw data was assessed using FastQC v.0.11.5 software [25]. Adapters and low-quality bases (Phred score less than 20) were removed by the BBDuk program with the BBtools package [26]. After data trimming, the genome assembly was performed by SPAdes v.3.11.1 tool [27]. The assembly data was checked in QUAST, and the presence of single-copy orthologs was verified with BUSCO. For genome annotation, MAKER2 v.2.31.9 [28] with the SNAP predictor v.2006-07-28 program [29] was used to predict genes and proteins from the genome. Proteins of the order Hypocreales (629 species) were used to provide evidence of protein homology.

### 2.4. Functional Annotation of the O. australis (CCMB661) Genome

After structural annotation, the functional characterization was performed by the GoFeat tool [30], using the following databases: UNIPROT [31]; INTERPRO [32]; PFAM [33]; NCBI [34]; EMBL [35]; KEGG [36]; and Gene Ontology [37].

### 2.5. Analysis of Predicted Secretome

For the characterization of the secreted protein domains, the database of protein families (Pfam) and the hidden Markov model (HMM) profile, which detects similarity between sequences, were used [38,39]. Additionally, we used the platform SECRETOOL, which allows the prediction of the subcellular location, which is one of the main aspects adopted for the definition of its functions [40,41].

### 2.6. In Silico Characterization in Plant Biomass Degradation

The CAZymes of *O. australis* CCMB661 were functionally annotated through the dbCAN v2 webserver, considering the genes encoding plant biomass-degrading enzymes predicted by the MAKER2 program [42]. For this analysis, the HMMER and DIAMOND tools were selected. Thus, the expected results in both were considered, increasing the reliability of our data.

### 2.7. Phylogenetic Analyses

In order to confirm the fungal identification and the placement of *O. australis* compared to different species of the genus *Ophiocordyceps*, we sequenced the internal transcribed spacer (ITS) genomic region from the isolate *O. australis* CCMB661 using the primer pair ITS1 and ITS4 [43]. The large ribosomal subunit (LSU) and the translation elongation factor (TEF) regions were obtained from the whole genome data from the same isolate, searching for the sequences of the primer pairs LR0R and LR5 (for LSU) [44], and 2218R and 983F (for TEF) [45], using the online PCR tool [46]. Sequences from our isolate were compared to homologous sequences using the NCBI nucleotide database BLASTn to ensure that all the sequences were from the genus *Ophiocordyceps*. We also included sequences from other studies (Table S1) that were selected from the GenBank (NCBI) database in our phylogenetic analyzes. Sequences from previous studies were determined by analyzing each *Ophiocordyceps* species that had all those three genomic regions deposited, avoiding sequences that were too divergent. After sequence selection, the final dataset consisted of 31 ITS, 34 LSU, and 34 TEF sequences. Individual gene alignments were generated by Geneious Prime 2020.0.4 [47]. The alignment of every gene was improved manually and concatenated into a single combined dataset. Gaps were treated as missing data, and ambiguously aligned regions were excluded from phylogenetic analyzes. The final alignment length was 2.836 bp: 897 bp for ITS, 949 for LSU, and 990 bp for TEF. Maximum Likelihood analyzes were performed with RAxML HPC-BlackBox [48] to reconstruct single trees for each region and the concatenated phylogenetic tree of ITS, LSU, and TEF. For all the analyzes, the nucleotide substitution model was established based on the Akaike information criterion (AIC) and likelihood ratio of jModelTest2 v 2.1.10 [49] on CIPRES Science Gateway v 3.3 [50]. Maximum Likelihood performed with the concatenated dataset containing the three loci consisted of five data partitions, including one for ITS, one for LSU, and three for the coding region TEF. The GTR-GAMMA nucleotide substitution model was employed to generate 1000 bootstrap replicates. Phylogenetic trees were visualized using FigTree v. 1.4.3 [51] and edited using Inkscape [52]. The species *Metacordyceps taii* (Hypocreales, Clavicipitaceae) was used as outgroup [53]. Parasitic fungi included in our analyzes were isolated from different arthropod hosts. We indicated each arthropod order with one icon based on information available in original papers in which the respective fungal sequences were published (Table S1).

## 3. Results

### 3.1. Genome Assembly

The whole-genome sequence has been deposited at DDBJ/ENA/GenBank under the accession number JACJUF000000000. The version analyzed in this paper is version JACJUF010000000. The assembled (Table 1) and annotated genome was compared with the reference genome of *O. australis* strain Map64 (accession number: PRJNA388689). The genomic data completeness in terms of expected gene content and single-copy orthologs indicated that our isolate CCMB661 is similar to the reference genome *O. australis* Map64 (Figure S1), which displayed 8,043 predicted genes that code for proteins using MAKER2. 

### 3.2. Predicted Secretome

**Figure 1:**
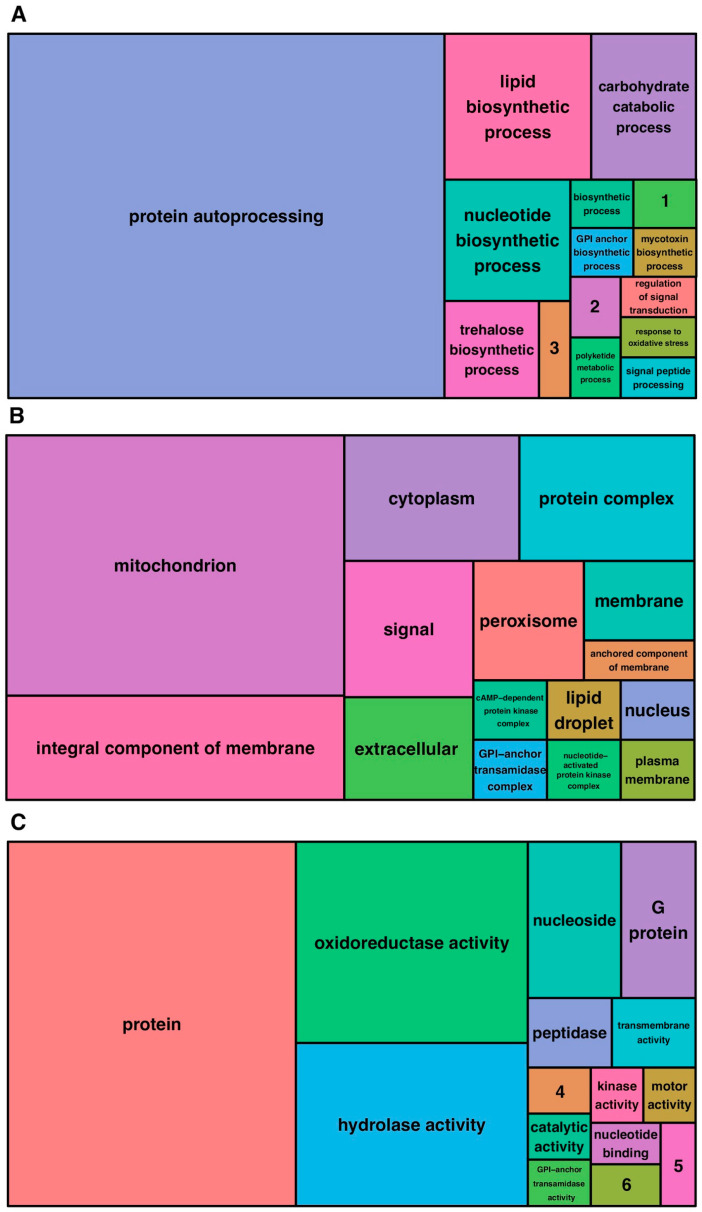
GOFeat annotation: Distinct identified genes proportionally displayed by main Gene Ontology categories. (**A**) Biological process. (**B**) Cellular component. (**C**) Molecular function. 1 aflatoxin biosynthetic process. 2 G protein-coupled receptor signaling pathway. 3 pathogenesis. 4 alpha,alpha-trehalose-phosphate synthase (UDP-forming) activity. 5 signal recognition particle binding. 6 triglyceride lipase activity.

A total of 8043 proteins were predicted for the fungus *O. australis* CCM661, of which 4,435 (55.14%) were not characterized. The GoFeat analysis classified these proteins into three groups according to gene ontologies: biological processes (23.34%), cellular components (27.80%), and molecular functions (48.86%). The category “biological processes” refers to molecular events related to cellular functioning; the category “cellular components” to terms associated with their intra- or extracellular location; and “molecular functions” circumscribes elementary activities of the products of genes at the molecular level. Among the biological processes, the ones that were most relevant to the genome studied were the carbohydrate pathways (5), lipid pathways (7), nucleotide pathways (5), protein pathways (52), and trehalose pathways (3) (Figure 1A). Whereas, considering the cellular components, those that were associated with more processes were: cytoplasm (5), integral membrane component (8), mitochondria (20), protein complex (5), and signal (4) (Figure 1B). For molecular functions, the most representative were G protein (4), hydrolase activity (13), nucleoside (5), oxidoreductase activity (16), and protein (36), as shown in Figure 1C.

### 3.3. Enzymes Involved in the Infection Process

#### 3.3.1. Adhesion and Recognition

**Figure 2:**
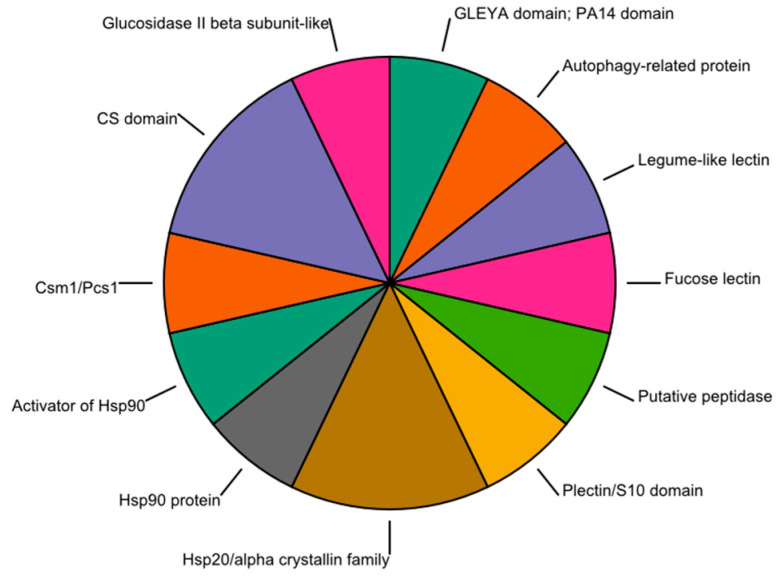
Enzymes directly involved in the adhesion and recognition stages of the infection process of the insect (ant) by the fungus. Each sector proportionally represents distinct groups (based on their specific protein domains) of potentially secreted enzymes.

In the present study, at least 14 proteins appear to be related to the processes of adhesion and recognition of the host by the fungus. Among them, four have lectin-like domains (gleya, PA14, Legume-like lectin family, Legume lectin domain, and Fungal fucose-specific lectin). One predicted domain corresponds to the binding protein domain (plectin/S10 domain), two belong to the peptidase MA clan (Putative peptidase family and Glucosidase II beta subunit-like protein), and all the others are associated with hydrophobins: hsp1 and hsp2 (Figure 2).

The lectin domains showed location prediction related to cellular components (extracellular space—GO: 0005615, endomembrane system—GO: 0012505, plasma membrane—GO:0005886) and distinct characteristics (signal peptide and Helix transmembrane). On the other hand, the HSP90 domain presented protein folding (GO: 0006457) as a biological process and as molecular functions, ATP binding (GO: 0005524) and unfolded protein binding (GO: 0051082), with the cytoplasm (GO: 0005737) as a prediction of cell location. Another 48 binding proteins containing domain J were found in the *O. australis* CCMB661 genome.

#### 3.3.2. Germination

Four proteins could be related to the germination stage, two of which have the HORMA domain and belong to the Clan Mad2 superfamily (CL0651). Another protein was characterized based on our bioinformatics analysis by the presence of the following domains: Mad3/BUB1 homology region 1 (PF08311), Mad3/BUB1 homology region 2 (PF08171), and Protein kinase domain (PF00069), and the fourth protein displayed only the Mitotic checkpoint protein domain (PF05557). Three proteins showed mitotic spindle assembly checkpoint (GO: 0007094) as a biological process, except for the protein that contains the kinase domain, which also exhibited protein phosphorylation (GO: 0006468) as a biological process and as molecular functions: ATP binding (GO: 0005524) and protein kinase activity (GO: 0004672). Regarding the prediction of cell localization, three of these proteins were predicted in the cytoplasm (GO: 0005737) and one, containing the Mad domain, in the mitochondrion (GO: 0005739), with a predicted feature as mitochondrial transit peptide.

Still considering the kinase domain, it displayed two active sites with similarities described in PFAM (Q5RD27 and Q7TPK6). The first one, an SRSF protein kinase 1, is linked with cell cycle progression, cell differentiation, and intracellular signal transduction. The second, a serine/threonine-protein kinase WNK4, plays an important role in regulating the cell process, protein localization, and cellular signaling. These factors led us to believe that these proteins may be related to the germination process.

#### 3.3.3. Cuticle Degradation

##### Chitinases

At least 15 chitinases have been found in the *O. australis* CCMB661 genome, of which 11 had an active site predicted in PFAM, indicating similarity to Q9W092, probable chitinase 2 (Cht2). All these enzymes contain the domain Glycosyl hydrolases family 18—GH18, and in one of them, the domain of unknown function—DUF929 was also found, which was originally described from a family of archaeon *Sulfolobus* proteins. These enzymes are involved in the carbohydrate metabolic process (GO: 0005975), related to hydrolase activity, hydrolyzing O-glycosyl compounds (GO: 0004553), chitin-binding (GO: 0008061), and chitinase activity (GO: 0004568). As for the prediction of subcellular location, nine proteins were predicted to be located in the extracellular space (GO: 0005615), four are from in the cytoplasm (GO: 0005737), one from in the mitochondrion (GO: 0005739), and one anchored component of in the plasma membrane (GO: 0046658), having predicted characteristics, in its majority, to signal peptides and, in one case, to the lipidation GPI-anchor. Another Glycosyl hydrolase domain associated with pathogenicity was also found in the studied genome (GH16—Glucanase), displayed in 12 proteins.

##### Proteases and Associated Domains

A total of 25 proteins were annotated, with 23 corresponding to protease domains, one to the LysM domain, and another to the Glycosyl hydrolase family 61—GH61. Among proteases, nine contained the Subtilase Family (Pr1) domain, 13 were aminopeptidases, and one was a cysteine protease (Pr4) (Table S2). 

Eight predicted Subtilase Family (Pr1) domains exhibited proteolysis (GO: 0006508) as a biological process and molecular function serine-type endopeptidase activity (GO: 0004252), and one predicted domain chitin-binding (GO: 000806) as a molecular function, besides containing the additional domain chitin recognition protein. Furthermore, most of these proteins exhibited, as a prediction subcellular location, the extracellular space (GO: 0005615), except for the one that also showed the proprotein convertase P-domain, which was predicted as subcellular location component integral of the membrane (GO: 0016021) and plasma membrane (GO: 0005886), and it is considered an alpha-helix transmembrane domain. In addition, the nine domains of Subtilase Family (Pr1) have an active site predicted by PFAM and similarity with the subtilisin-like protease SBT4.5 (F4JA91), two of them also showed similarity with peptidase S53 domain-containing protein, and one of them also showed similarity with the protein subtilisin-like protease 12, gene SUB12 (D4AQA9). The function of the SUB12 gene is the secretion of a subtilisin-like protease with keratinolytic activity, which contributes to pathogenicity. Thus, this subtilisin-like protease 12 showed pathogenesis (GO: 0009405) as a biological process [54].

The peptidase family C54 domain displayed, as biological processes, protein transport (GO: 0015031) and autophagy (GO: 0006914); as molecular function cysteine-type peptidase activity (GO: 0008234); and subcellular location in the nucleus (GO: 0005634) and cytoplasm (GO: 0005737).

Considering the aminopeptidases, the two that have the domain Dipeptidyl peptidase IV (DPP IV) N-terminal region showed an active site predicted by PFAM and similarity to probable dipeptidyl peptidase 4, gene DPP4 (D4APE2). This protein is responsible for removing N-terminal dipeptides sequentially from polypeptides having unsubstituted N-termini, provided that the penultimate residue is proline, contributing to pathogenicity [55].

##### Phospholipases, P450, and Lipid Droplet

At least 19 proteins possibly suggesting an association with lipid degradation have been annotated for the genome of *O. australis* CCMB661. Among these, 13 were of the phospholipase type, and two contained the Beta-lactamase superfamily domain, with molecular function N-acylphosphatidylethanolamine-specific phospholipase D activity; two predicted proteins are related to cytochrome P450 (flavodoxin, cytochrome P450, FAD-binding, and oxidoreductase NAD-binding domain); and two displayed lipid droplet as cellular component (squalene-hopene cyclase C-terminal and squalene-hopene cyclase N-terminal domain). Importantly, these squalene-hopene cyclase domains were similar to the active site (Pfam Q4WR16) protostadienol synthase helA, a protein involved in the biosynthesis of mycotoxins in *Aspergillus fumigatus* [56].

Among the phospholipases (Table S3), three had the Patatin-like phospholipase domain; four exhibited Phosphatidylinositol-specific phospholipase C, X domain, and Phosphatidylinositol-specific phospholipase C, Y domain; two presented the Phospholipase D Active site motif domain; two were of the type A2 (Prokaryotic phospholipase A2 and Platelet-activating factor acetylhydrolase, isoform II); and two were Lysophospholipase catalytic domain, which showed similarity to the active site (Pfam Q86XP0), cytosolic phospholipase A2 delta, calcium-dependent that selectively hydrolyzes glycerophospholipids in the sn-2 position [57].

### 3.4. Other Important Proteins for the Infection Process

In the *O. australis* genome, 113 MFS and four ABC transporters, five mycotoxins, 29 enterotoxins, 12 protein tyrosine phosphatase—PTP, and one ubiquitin-activating enzyme E1-like were identified, which are also considered essential domains for understanding the infection process and pathogenesis, in addition to the biology of the species.

#### 3.4.1. Enterotoxins

**Figure 3:**
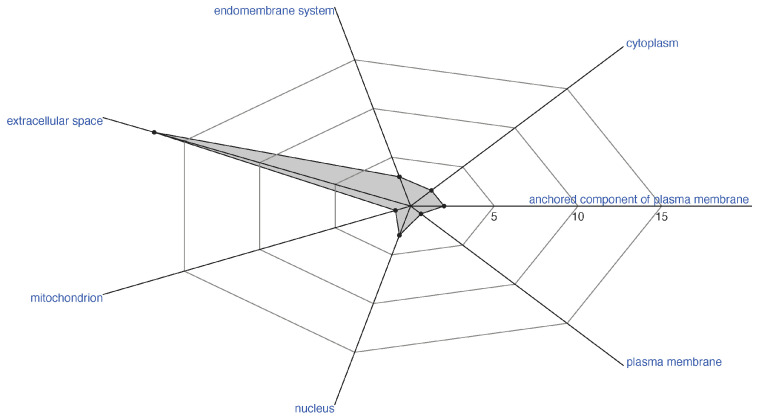
Distinct subcellular location of detected enterotoxins of the entomopathogenic fungi *Ophiocordyceps australis*. Dots and shadowed areas in the radar plot proportionally depict the number of enterotoxins in each subcellular location.

A total of 33 proteins related to pathogenesis (GO: 0009405) were found, among which 29 were enterotoxins, three were Kp4 domain-containing proteins, and one was an uncharacterized protein. Amongst the enterotoxins, all comprised the heat-labile enterotoxin alpha chain domain and are categorized as toxin activity (GO: 0090729), except for one, which has the domain Pertussis toxin, subunit 1 with NAD+ ADP-ribosyltransferase activity (GO: 0003950). Regarding the prediction of the subcellular location, most of the enterotoxins (17) are from the extracellular space (GO: 0005615), presenting the signal peptide as a predicted characteristic (Figure 3). Moreover, it is worth noting that the two enterotoxins that exhibited a cellular component anchored component of the plasma membrane (GO: 004665) have as predicted characteristics both signal peptide and lipidation GPI-anchor.

#### 3.4.2. Protein Tyrosine Phosphatases—PTPs

Out of the 12 PTPs, initially, six were not characterized; after annotation in the PFAM, it was possible to identify five domains, as can be seen in Table S4. Most of the PTP domains showed protein dephosphorylation (GO: 0006470) and/or dephosphorylation (GO: 0016311) as a biological process, as molecular function protein tyrosine/serine/threonine phosphatase activity (GO: 0008138) and/or protein tyrosine phosphatase activity (GO: 0004725), and as a prediction of cytoplasm (GO: 0005737) cell location. Additionally, two proteins that contained the protein-tyrosine phosphatase domain (PF00102) had the predicted characteristic transmembrane alpha-helix, while the uncharacterized protein showed mitochondrial transit peptide as a characteristic.

### 3.5. Enzymes Associated with Plant Biomass Degradation

A total of 361 genes encoding CAZymes were predicted, of which 175 genes were confirmed for both prediction tools (HMMER and DIAMOND). Among these genes, 28 are responsible for encoding cellulose, hemicellulose (xylan, xyloglucan, and galactomannan), pectin, starch, and inulin (Table 2). These results corroborate our hypothesis that *O. australis* acts as parasitizing ants and can also be considered endophytic, completing some stage of their life cycle associated with plants, probably to have access to their host. All these evaluated enzymes belong to GHs family and did not display a signal peptide structure. Thus, the GHs that most occurred in our study were: GH2 (4), GH3 (6), GH5 (4), and GH31 (3) (Table 2). Moreover, a search performed by our research group in two metagenomic databases (BioSample—NCBI and MG-RAST) also pointed to the association of *Ophiocordyceps* species with plants in 17 samples, mainly for *O. sinensis* parasites of larvae of moth (15) and *O. unilateralis* ant parasite (7) (data not shown).

### 3.6. Phylogenetic Analyses

**Figure 4:**
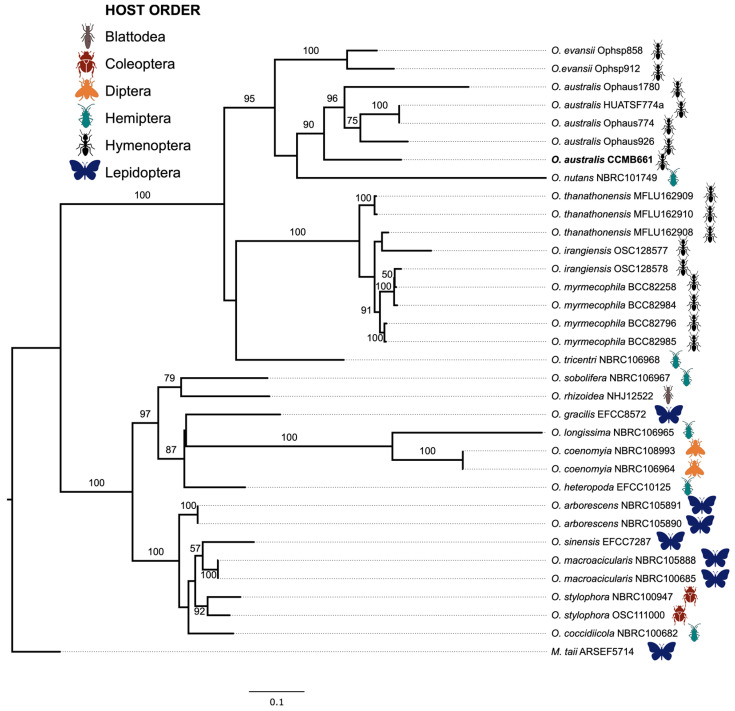
Phylogenetic tree showing the position of the isolate *Ophiocordyceps australis* CCMB661 obtained parasitizing the ant *Neoponera curvinodis* Forel, 1899, compared to different species of the genus *Ophiocordyceps*. Analysis was based on concatenated sequences of ITS, LSU, and TEF markers. Maximum Likelihood (RAxML) tree was built using the GTR-GAMMA nucleotide substitution model, and the values on branches indicate the percentage of bootstrap based on 1.000 replicates. Bars show nucleotide substitutions per site. The tree includes a total of 31 ITS, 34 LSU, and 34 TEF sequences. We included 33 *Ophiocordyceps* sequences from previous studies obtained from GenBank. The final alignment had 2.836 base pairs. Species from GenBank are followed by their strain codes. For each sequence included in this phylogenetic analysis, we show the arthropod host order from which fungi were isolated (Blattodea = brown, Coleoptera = red, Diptera = orange, Hemiptera = green, Hymenoptera = black, Lepidoptera = blue). The fungus *Metacordyceps taii* was used as outgroup.

The ITS sequences of *O. australis* CCMB661 as well as of the isolates named *O. australis* previously selected in GenBank formed a monophyletic clade (Figure 4). We found out that, among the 33 sequences of *Ophiocordyceps* included in the analysis, 18 species of the genus were represented. Sixteen specimens belong to five fungal species associated with Hymenoptera hosts, including our isolate (*O. australis*, *O. evansii*, *O. irangiensis*, *O. myrmecophila*, *O. thanathonensis*). Additionally, six species with Hemiptera hosts (*O. coccidiicola*, *O. heteropoda*, *O. longissima*, *O. nutans*, *O. sobolifera*, and *O. tricentri*), and four species were associated with Lepidoptera (*O. arborescens*, *O. gracilis*, *O. macroacicularis*, and *O. sinensis*). The species *O. rhizoidea*, *O. stylophora,* and *O. coenomyia*, associated with Blattodea, Coleoptera, and Diptera, respectively, were also recovered in the analysis.

## 4. Discussion

In this study, the entomopathogenic fungus *O. australis* was collected from Atlantic Forest ecosystems parasitizing the ant *N. curvinodis* Forel, 1899. This is the first report of *O. australis* associated with the host species *N. curvinodis*, extending the host range of this fungal species. Nevertheless, former studies documented that *O. australis* was found parasitizing another host species, such as *Pachycondyla striata* [58] in Atlantic Forest in Brazil, and *Paraponera clavata* and *P. crassinoda* in the Amazon region of Colombia [59]. Evans [60] described *O. australis* in West Africa, parasitizing exclusively *Paltothyreus tarsatus*. The same author also pointed out that in the forests of South America (Ecuador and Brazil), *O. australis* usually occurs in ants included in the genus *Pachycondila*. Sanjuan et al. [17] addressed new entomopathogenic fungi from the Amazon and reported that *O. australis* parasitizing the adult ant *P. crassinoda*. In a more recent study [19], however, 48 specimens of *O. australis* collected at Reserva Ducke (Central Amazon, Brazil) were found parasitizing the following host ants: *Neoponera cavinodis*, *N. crenata*, *N. foetida*, *N. unidentata*, *N. villosa*, *Odontomachus hastatus*, *Pachycondyla impressa*, *Pachycondyla sp*. These results reveal a high diversity of potential hosts for *O. australis* and its preference for ants Ponerinae, Ponerini, indicating that *O. australis* is not a specific parasite at the species level.

### 4.1. Predicted Secretome

Some biological processes are directly related to the host-parasite interaction, which can evidence the main steps to elucidate how infection processes occur. Thus, it is essential to highlight that the insect cuticle is composed of lipids, proteins, and carbohydrates (chitin), which explains the need for the fungus to mobilize the pathways of these compounds for penetrating the host and, later, assimilating during the stages of growth and sporulation on the corpse. Pathways of lipid assimilation by exogenous substrates are essential for a successful infection to occur, and lipid growth substrates have significant effects on the virulence of fungal infection propagules, such as conidia. In addition, the production of lipases plays a crucial role in the breakdown of cuticles and pathways associated with triglyceride metabolism and phospholipid homeostasis contribute to the host invasion [61]. Nucleotides and nucleosides are chemical constituents and bioactive components in *Cordyceps* and *Ophiocordyceps* [62,63]. As highlighted in Chiriví et al. [64], nucleosides are involved in regulating and modulating several derived biological processes. It is important to note that arthropod parasites can use trehalose as a carbon source, a molecule abundant in the tissues of these animals, especially in their hemolymph [12,65].

In eukaryotes, most of the proteins are synthesized in the cytosol and are directed to different subcellular locations. Therefore, extracellular proteins contain an N-terminal sequence, the signal peptide, which is recognized by secretory pathways and mediates these proteins to the endoplasmic reticulum for transportation [40]. According to Dönnes and Höglund [66], the subcellular location of a given protein is specific, as well as crucial for the good performance of its function. Thus, 34% of the proteins located in the nucleus are associated with the transcription process, and others are considered essential for spore viability. Meanwhile, 26% of the proteins found in the mitochondria are responsible for the cell respiration process [67,68].

In entomopathogenic fungi, oxidoreductase enzymes are important as a defense mechanism against the host insect’s immune system, which, during the infection, secretes reactive oxygen species (ROS) in an attempt to kill the pathogen. In response, the fungus produces these enzymes that carry out redox reactions, such as peroxidases that act in the elimination of ROS [69]. In this context, de Bekker et al. [22] highlighted that oxidation-reduction reactions interfere with parasite-host interactions, more specifically in the parasite redox biology. Thus, the host’s immune responses include oxidative attacks, and the production of antioxidant enzymes by the parasite is considered essential for virulence and maintenance of the infections. Therefore, oxidation-reduction seems to be a crucial mechanism for host-parasite interaction. Furthermore, for Vongsangnak et al. [70], the presence of multiple hydrolytic enzymes in *C. militaris*, such as proteases and lipases, plays a role in growth, development, and survival in their natural habitat. In addition, hydrolases are also associated with chitin mineralization [71].

### 4.2. Proteins and Enzymes Involved in the Infection Process

#### 4.2.1. Adhesion and Recognition

The fungal spore adheres to the host cuticle in the adhesion stage, requiring electrostatic mechanisms and connection to specific receptors for proper fixation. The main enzymes involved in this process include adhesins, lectins, and hydrophobins (hsp1 and hsp2) [6,11,72]. Lectins are non-immune glycoproteins that bind to specific carbohydrates and are related to recognition events at the cellular and molecular levels. The Gleya domain is found in fungal adhesins and resembles the lectin-like domain found in the proteins Flo and Epa of *Saccharomyces cerevisiae* and *Candida glabrata*, respectively. Adhesins that have this domain have lost GPI anchors (glycosylphosphatidylinositol) and display a specific terminal region (signal peptide) and the repeated conserved sequence G(M/L)(E/A/N/Q)YA. The PA14 domain, also compatible with carbohydrate binding, can be found in glycosidases, such as beta-glucosidases in bacteria, glycosyltransferases, proteases, amidases, yeast adhesins, and bacterial toxins, such as anthrax protective antigen (PA). The fucose-specific lectin fungal domain recognizes fucosylated glycans [73,74,75,76,77]. 

De Bekker et al. [22] mentioned that lectins bind to specific carbohydrates and mediate recognition in the host-parasite interaction. In this gene expression study related to pathogenicity in *O. unilateralis* s.l., two ricin-type lectins, one fucose-specific lectin, and one lectin-like flocculation protein were found. Lectins are extensively studied due to their therapeutic and biotechnological potential. For Reyes-Montaño and Vega-Castro [73], lectins, depending on their carbohydrate specificity, may have different biological activities and have already been evaluated for their potential as mitogenic agents, biomarkers, insecticides, and cytotoxic. Furthermore, they stated that the lectin legume domain has the highest potential as an insecticide or insectstatic activity, given the pattern of glycolysis in insect midgut cells. In this perspective, Macedo et al. [78] pointed out that lectins have been used as biotechnological tools for pest control.

According to Butt et al. [79], hydrophobins facilitate the fixation of conidia on hydrophobic surfaces. Thus, it mediates adhesion due to its similarity with the host hydrophobic epicuticle, forming hydrophobic interactions [9]. For the genome of *O. unilateralis* s.l., two hydrophobic surface binding proteins (HsbA) and one hydrophobin domain-containing protein were found [22]; however, for the specimen *O. australis* CCMB661 studied, the presence of these domains in its genome was not detected, but other proteins associated with Heat Shock Proteins (HSPs).

The presence of HSPs can be explained in *O. australis* CCMB661, along with other defense strategies, because entomopathogenic fungi in all the stages of infection are exposed to many stress conditions. Hence, these proteins act as chaperones, promoting the refolding of proteins. HSPs are highly conserved and ubiquitous and are known for their high capacity to respond to various stress conditions, such as extreme temperature, desiccation, anoxia, hypertonic stress, ultraviolet radiation, heavy metals, and alcohol, among others. Additionally, some HSPs are important in growth-related processes, including cell division and DNA synthesis. In *Cordyceps sinensis*, Hsp 70 and Hsp 90 are generally in high expression in the ascoma, so they may be related to their formation and development [10,80,81]. According to Prodromou [82], Hsp 90 implies several biological processes dependent on ATP.

#### 4.2.2. Germination

Infective spores adhere to the cuticle and germinate to form the infection structure (appressorium) and penetrate the insect epicuticle [20,72]. Mitotic arrest deficient (MAD) is a domain present in eukaryotes that consists of the mitotic checkpoint. In *S. cerevisiae* the Mad1-dependent complex comprises Mad2, Mad3, Bub3 and Cdc20 [83]. The HORMA domain is a highly conserved multifunctional protein-protein interaction module that can be found in several signaling pathways, such as spindle assembly checkpoint, recombination and DNA repair pathways, and autophagy initiation. These proteins occupy the main signaling junctions, working through the assembly and disassembly of the signal complex. The best-characterized protein in the HORMA domain is Mad2, an essential mediator of the spindle assembly checkpoint [84,85].

Mad1 and Mad2 in entomopathogenic fungi are widely attributed to adhesive properties [11,20,79,86]. Nonetheless, Wang and Leger [87] demonstrated that the expression of Mad1 and Mad2 of the entomopathogenic fungus *Metarhizium anisopliae* also interferes in the germination of the conidium, in the formation of the blastospore and the differentiation of the hyphal bodies in the insect. Mad1 is required to organize the cytoskeleton and cellular division, thus interfering in the cell cycle, and its interruption can delay germination. Moreover, for Mad1 and Mad2 to function as adhesins, they may need to be located on the cell surface. Therefore, this indicates that the Mad genes present in the genome of the species of *O. australis* CCMB661 may be involved in the germination and non-adherence process since they displayed prediction of the subcellular location in mitochondria and cytoplasm. Still, in this sense, Lovett and Leger [9] pointed out that the adhesin-like Mad1 protein mediates spore fixation, germ tube growth, and appressorium formation. For Ortiz-Urquiza and Keyhani [88], the loss of Mad1 implies reduced adhesion to the insect cuticle, germination, blastospore production, and virulence.

#### 4.2.3. Cuticle Degradation

**Figure 5:**
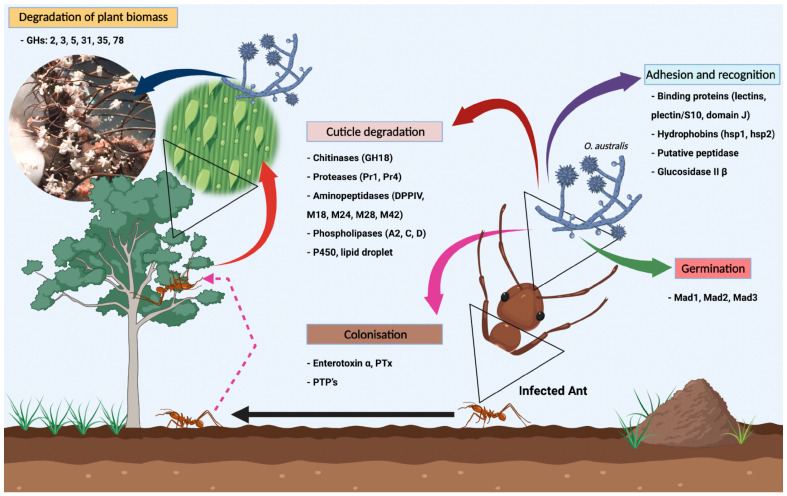
A general overview of the life cycle of the entomopathogenic fungus *O. australis* (CCMB661), emphasizing all the fungal infection processes in the ant *Neoponera curvinodis* Forel, 1899. The proteins related to each stage (Adhesion and Recognition), Germination, Cuticle Degradation, and Colonization) are listed.

Entomopathogenic fungi of the order Hypocreales usually infect their hosts through penetration of the cuticle. The key enzymes involved in this process include proteases, lipases, and chitinases (Figure 5) [12,89].

Chitinases are widely distributed in nature and have several functions in physiological processes, including tissue degradation and remodeling, nutrition by absorption, invasion and pathogenesis, and regulation of the immune response [90]. Glycoside hydrolases (EC 3.2.1.-) are a broad group of enzymes that hydrolyze the glycosidic chain between two or more carbohydrates or between a carbohydrate and a non-carbohydrate portion [91]. Glycoside hydrolase family 18 (GH18) chitinases (EC 3.2.1.14) catalyzes the biodegradation of the β-1,4 glycosidic chain into amino acids polysaccharides, such as chitin and chitooligosaccharides. In the study of the genome of *Hirsutella sinensis*, a total of 23 chitinases (endochitinases and exochitinases) were identified as associated with the infection process [62]. Additionally, Kobmoo et al. [12], studying the genome of *Ophicordyceps* zombie-ant fungi, including *O. australis*, highlighted 11 chitinases (GH18). Chitinases from subgroup B have a significant role in infections by entomopathogenic fungi. For instance, a study with the fungus *Metarhizium anisopliae* demonstrated that the overexpression of Chi2 showed high efficiency and, consequently, the death of the host since the suppression of this endochitinase caused a decrease in virulence for insects [92].

Proteases (EC3.4) are enzymes in the group of hydrolases responsible for catalyzing the hydrolysis reaction of protein-peptide bonds, and they may also show activity on ester and amide bonds. These enzymes are considered very important in the infectious process, in which subtilisin-like serine-protease Pr1 and trypsin-like protease Pr2 are the most studied because they are secreted during the first stage of cuticle degradation. Similarly, Zibaee and Ramzi [93] mentioned that Pr1 proteases are the most prevalent and effective for penetration into the cuticle, which can also be confirmed in our study. Serine proteases (EC 3.4.21) are endopeptidases characterized by the amino acid serine at the active site [94,95]. As highlighted in Vilcinskas [96], these fungal proteinases are recognized as virulence factors, as they facilitate penetration into the exoskeleton for later use of host proteins for nutrition, suppression of host defense cells, as well as the degradation of host defense molecules, specifically through the action of metalloproteinases. On the other hand, aminopeptidases and exopeptidases degrade proteins solubilized into amino acids to provide nutrients for entomopathogenic fungi [95]. 

A total of 26 proteases were found in the *H. sinensis* genome [62], including serine protease, arginase, cysteine protease and cuticle-degrading protease. In the study of de Bekker et al. [22], at least nine proteases were found in the genome of *O. unilateralis* s.l., related to manipulating bite behavior: six serine proteases (1 tripeptidyl-peptidase and five subtilisins), two aspartyl proteases, and one metallocarboxypeptidase. These proteases are generally secreted in pathogenic fungi, but their function and importance will vary according to the species, and they are widely recognized as virulence factors [22]. Kobmoo et al. [12] also mentioned that proteases facilitate cuticle penetration and virulence factors for host infection.

The conserved domain peptidase inhibitor I9 is a prodomain of self-inhibitory subtilases, which maintains the state of inactive zymogen and prevents the substrate from accessing the active site. It is an N-terminal propeptide domain of peptidases included in the S8A family of the MEROPS classification. It is also a type of subtilase responsible for modulating folding and pro-enzyme activity [97,98,99]. Besides the subtilases, the genome of *O. australis* CCMB661 has one cysteine endoprotease (Pr4). This protease is presented as a biological process autophagy, highlighted in Liu et al. [99], which is a ubiquitous process of degradation and recycling of resulting macromolecules in all eukaryotic cells. According to Koblitz [94], cysteine-proteases, also named sulfhydryl proteases (EC 3.4.22), have the amino acids cysteine and histidine conjugated in their active site. Tiago and Furlaneto [100] pointed out that Pr4 shows greater degradation activity of the cuticle when compared with Pr2; however, the role of these enzymes in parasitism is not well understood. For Butt et al. [79], subtilisin (Pr1), trypsin (Pr2), and cysteine (Pr4) are proteases associated with cuticle degradation in *Beauveria* and *Metarhizium* species.

The LysM domain is recognized for presenting virulence and pathogenicity factors in various plant-pathogen systems during infection [98]. Although, the LysM domain is also associated with the virulence of the fungus *Beauveria bassiana* due to its ability to evade immune responses in a host insect [101]. Lysin motifs (LysM) bind to peptidoglycan, chitin, and their derivatives. The LysM fraction can be found in several proteins, including chitinases, peptidases, receptor-like kinases, and effectors. This domain, both in plant pathogens and in entomopathogens, acts in the elimination of chitin oligosaccharides and in the host defense mechanisms. In addition, LysM proteins can protect the fungus cell wall against hydrolytic enzymes, including chitinases [102,103]. When binding to chitin oligomers of host immune receptors, LysM proteins secreted by fungi disturb the elicitation of host immunity triggered by chitin [104]. In the study of comparison of transcriptomes of fungi *Ophiocordyceps* that parasitize ants developed by Will and collaborators [105], the presence of the LysM domain was also detected, corroborating our results for the genome of *O. australis* CCMB661.

Lipases, including phospholipases, are essential enzymes for lipid degradation of the insect epicuticle and, together with cytochrome P450-specific enzymes, act in the assimilation of host external and internal lipid substrates during the proliferation of the fungus in the hemocoel [9,61,88,95]. Furthermore, fungal lipid droplets and their related enzymes (perilipin) act in the storage and mobilization of internal lipids and contribute to the success of the infection due to the increase in lipolysis, participating in practically all stages: penetration, immune evasion, and successful sporulation in host corpse [20,61].

Phospholipases are ubiquitous enzymes associated with several processes, such as membrane homeostasis, nutrient acquisition, and generation of bioactive molecules, which are crucial for microbial and fungal pathogenesis and virulence. These enzymes hydrolyze phospholipids into fatty acids and other lipophilic substances and are also involved in hyphae development. Moreover, they may be related to signaling messengers to induce stress tolerance and host immune responses in fungi [106,107]. Among the phospholipases, type A2 (PLA2) is considered the main one during the interaction of the fungus-host, since during the infection process, these enzymes are secreted and act not only in the acquisition of nutrients but also allowing the invasion of host tissues [106]. Also, La Camera et al. [108] indicated that genes and proteins related to the patatin domain (PLP2) phospholipases acyl hydrolases have a lipolytic activity that pathogens can exploit with different lifestyles to facilitate host colonization. Qi et al. [107] also highlighted that VdPLP, Patatin-Like Phospholipase, contributes to penetration and colonization of the phytopathogenic fungus *Verticillium dahliae* in roots of *Nicotiana benthamiana*, which might be required for pathogenicity.

According to Jin et al. [62], the genome of *H. sinensis* has about 65 phospholipases (A1, A2, B, C, and D) involved in the infection mechanism. Amongst all these phospholipase classes, only types A1 and B were not predicted for the genome of *O. australis* CCMB661, with a predominance of type A2, which presented as biological processes lipid metabolic process, lipid catabolic process, phospholipid metabolic process, phospholipid catabolic process, and arachidonic acid secretion.

### 4.3. Other Important Proteins for the Infection Process

#### 4.3.1. Enterotoxins

Heat-labile enterotoxins are small, secreted proteins, critical effectors for the adaptation and co-evolution of the host in *Ophiocordyceps*. In three complexes of species that parasitize ants, *O. unilateralis*, *O. australis*, and *O. subramanianii*, the presence of these enterotoxins suggested an enrichment of functions associated with pathogenesis [12]. Heat-labile enterotoxin is an AB5 toxin that consists of an active catalytic site, subunit A, linked to five non-toxic B subunits arranged in a homopentamer. Pentamer B has the function of binding membranes [109,110]. According to de Bekker et al. [22], the genome of *O. unilateralis* s.l., presented 34 genes encoded proteins from the enterotoxin_a domain (PF01375), among which nine are homologs to cholera toxins. Additionally, de Bekker et al. [21] highlighted that these enterotoxins are important for *Ophiocordyceps* species that manipulate behavior since they interfere with host chemosignaling molecules, which can vary between 20 to 36 in species that parasitize ants. In the genome of *O. camponoti-floridani*, a total of 35 enterotoxin_a was identified. The high number of proteins with this enterotoxin domain suggests its central role in the pathogenesis of *Ophiocordyceps*, especially during host infection and manipulation [105].

Another enterotoxin domain found in the *O. australis* CCMB661 genome was Pertussis toxin, subunit 1 (PF02917). Pertussis toxin (PTx) is considered one of the main virulence factors produced by *Bordetella pertussis*, which plays a central role in the pathogenesis and infection. PTx has two domains: ADP-ribosyltransferase enzymatic subunit S1 (A-protomer) and the host cell binding carbohydrate-binding subunits S2–5 (B-oligomer) facilitates the entry of PTx into the cell. PTx inhibits the host protein G receptor, causing a range of effects. In Will et al. [105], the presence of pertussis toxin A was also detected in the genome of *O. camponoti-floridani* in the course of the infection. The active site of pertussis was also similar to heat-labile enterotoxins, and it interferes with the GPCR pathway [111,112]. 

Two enterotoxins displayed the complex membrane anchoring system, lipidation Glycosylphosphatidylinositol Anchor (GPI anchor). GPI anchor is a glycolipid structure, a post-translation modification, added to the C-terminus of many eukaryotic proteins that participate in diverse biological processes, including pathogenesis and signal transduction [113]. Kobmoo et al. [12] also mentioned that lipids are involved in a series of pathophysiological processes in pathogenic fungi.

#### 4.3.2. Protein Tyrosine Phosphatase—PTP

PTPs are crucial signal transduction enzymes that control cellular protein tyrosine phosphorylation levels. It consists of 3 groups of enzymes capable of dephosphorylating phosphorylated tyrosine residues: classical PTP, dual-specificity PTP, and low-molecular-weight PTP [114]. Bollu et al. [115] define PTP as a family of enzymes that hydrolytically remove phosphate groups from proteins.

According to Will et al. [105], enterotoxins and PTP interfere with host neuronal functions, such as neuronal maintenance and development, circadian rhythm, and sensitivity to light, smell, and memory. Nevertheless, the role of PTP in the manipulation of *Camponotus* ants is still uncertain, even though a study involving *O. camponoti-floridani* identified seven genes that code for PTP, among which five were regulated positively in the culture during the manipulation, and three supposedly secreted. Thus, these same authors suggest that the PTP of fungi could deregulate the activity of ants, changing their behavior and locomotor activity. In this sense, Houte et al. [116] also pointed out that PTP, in baculovirus, induces hyperactivity behavior in the host (caterpillars) through direct or indirect action in the host Kinase G (PKG) pathway and/or in the circadian cycle pathway. In de Bekker et al. [22], PTPs are also among the proteins secreted by *O. unilateralis* s.l., being related to the behavior manipulation of the host *C. castaneus* (ant), interfering in its locomotor activity.

### 4.4. Enzymes Associated with Plant Degradation

CAZymes are considered the most essential enzymes for the metabolism of complex carbohydrates, responsible for the breakdown, biosynthesis, or modification of glycoconjugates, oligosaccharides, and polysaccharides. CAZymes are organized into six main classes, of which the GHs are one of them, whose function is to hydrolyze the glycosidic chain between two or more carbohydrates or between a carbohydrate and a non-carbohydrate role, such as proteins and lipids. Among the main GHs found in fungi involved in plant biomass degradation are the families: GH2, GH3, GH5, GH27, GH31, GH35, GH43, GH74, and GH78 [13,42]. It is necessary to highlight that most enzymes mentioned above are present in the genome of *O. australis* CCMB661, except for three (GH27, GH43, and GH74), suggesting that this species developed different adaptations in its genome to perform as a plant pathogen [6]. Wichadakul et al. [117] agree that some GH and Polysaccharide lyase (PL) are adaptations in plant pathogens, including GH28 and GH78 present in *O. australis*. Among these GHs, the most frequently occurred in our study were: GH2, GH3, GH5, and GH31.

Hypocreales fungi, described only as entomopathogens, may also be capable of presenting an endophytic life cycle, such as *O. sinensis*, found in leaves or roots of 23 plant species facilitating the infection of the root-eating host larva [8]. Lei et al. [23] also mentioned that *O. sinensis* could colonize different tissues, both from *Thitarodes* larvae and plants, especially *Ranunculus tanguticus*, showing an interkingdom colonization potential in the development of this species of *Ophiocordyceps*.

Species of *Ophiocordyceps* are described as entomopathogenic and endophytic. The *O. crassispora* Rct61 strain was isolated from *Rhodiola crenulata* [118]. Still, in this perspective, Wu et al. [119] highlighted that *O. sinensis* might be associated with *P. anserina* roots, establishing an endophytic relationship with plants. Wichadakul et al. [117] stated that many genes found in the genome of *O. polyrhachis-furcata* are shared with other entomopathogenic fungi and plant pathogens. Furthermore, these same authors also suggested that the presence of Mad2 in entomopathogenic fungi could be related to the dual way of life of these fungi, parasitizing insects, and at the same time, having the ability to parasitize plants.

## 5. Conclusions

The present study allowed us to better understand the biology, genome, predicted secretome, and evolution of the entomopathogenic fungus *O. australis* and, especially, the molecular machinery that controls the infective process in the host-parasite interaction. Proteases (Pr1 and Pr4) and their inhibitors I9, chitinases (Cht2), lipases, and virulent toxins (enterotoxins and PTx) could facilitate the exploration of the use of this species as a model for better elucidation of the entomopathogenic process. Furthermore, this species could be considered generalist and is able to parasitize more than one group of hosts, contributing to interkingdom colonization. 

## Figures and Tables

**Table 1:** Genome assembly statistics for the *Ophiocordyceps australis* CCMB661 and Map64 genomes.

	CCMB661	Map64
Longest Scaffold	491,455	427,808
Number of contigs	774	594
Genome length	30,311,510	23,324,075
N50	92,624	112,405
L50	90	59
% GC	46.36	53.13

**Table 2:** Prediction of *Ophiocordyceps australis* CCMB661 genes involved in plant biomass degradation (from CAZy family) and their respective product information.

Sequence ID	CAZy Family	Product Information
NODE_200_length_47715 NODE_272_length_31825	GH1	Cellulose, hemicellulose (galactomannan)
NODE_113_length_83284 NODE_26_length_194473 NODE_28_length_188649 NODE_58_length_135625 NODE_63_length_123432 NODE_94_length_88402	GH3	Cellulose, hemicellulose (galactomannan, xylan), pectin
NODE_229_length_41116 NODE_327_length_25465 NODE_58_length_135625 NODE_5_length_312948	GH5	Cellulose, hemicellulose (galactomannan), pectin
NODE_186_length_51079 NODE_57_length_137212 NODE_62_length_124067	GH31	Hemicellulose (xyloglucan), starch
NODE_120_length_78160 NODE_152_length_64882 NODE_20_length_205932 NODE_275_length_30979	GH2	Hemicellulose (galactomannan, xylan, xyloglucan), pectin
NODE_196_length_48790	GH36	Hemicellulose (galactomannan, xylan, xyloglucan)
NODE_232_length_40978	GH28	Pectin
NODE_6_length_286604	GH35	Hemicellulose (galactomannan, xylan, xyloglucan), pectin
NODE_120_length_78160	GH78	Pectin
NODE_33_length_181024 NODE_82_length_100911	GH13	Starch
NODE_91_length_92211	GH15	Starch
NODE_521_length_6169	GH32	Inulin
NODE_58_length_135625	GH115	Hemicellulose (xylan)

## Data Availability

The genome data are available in the NCBI repository under the accession number JACJUF000000000 (https://www.ncbi.nlm.nih.gov/nuccore/JACJUF000000000—accessed on 31 December 2022).

## Supplementary Materials

The following supporting information can be downloaded at: https://www.mdpi.com/article/10.3390/jof9010110/s1, Figure S1: BUSCO quality assessment results for the genome of *Ophiocordyceps australis* CCMB661 and Map64; Table S1: Sequences of *Ophiocordyceps* species from NCBI-GenBank databases used in phylogenetic analyzes and their associated metadata; Table S2: Proteases and related domains; Table S3: Predicted phospholipase domains for the entomopathogenic fungus *O. australis* CCMB661; Table S4: Protein tyrosine phosphatase—PTP domains.
