# Supplementary material for: Unraveling the Secrets of a Double-Life Fungus by Genomics: Ophiocordyceps australis CCMB661 Displays Molecular Machinery for Both Parasitic and Endophytic Lifestyles
Source: J Fungi (Basel). 2023 Jan 13;9(1):110. doi: 10.3390/jof9010110 (PMC9864599; doi:10.3390/jof9010110)
Supplement: Supplementary file 1 [file jof-09-00110-s001.zip › jof-2114354-Supplementary material.pdf]

# Supplementary material

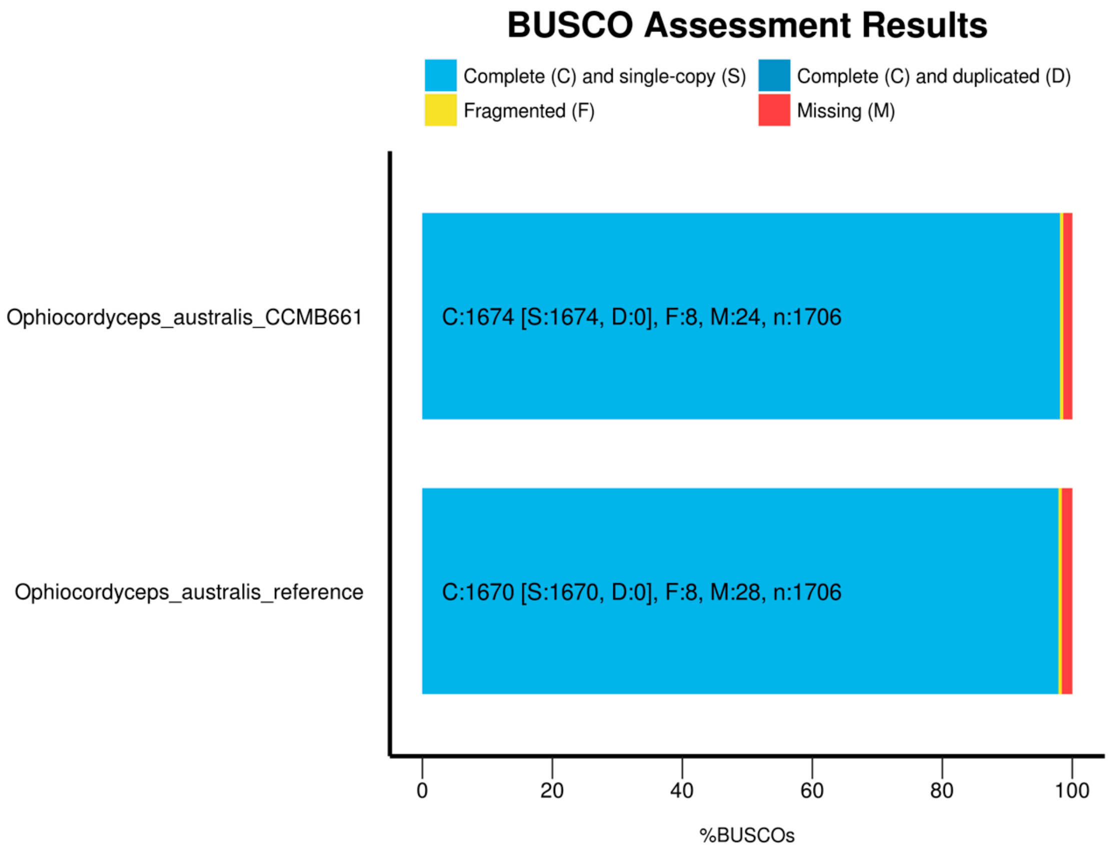


**Figure S1:** BUSCO quality assessment results for the genome of *Ophiocordyceps australis* CCMB661 and Map64.

.

**Table S1:** Sequences of *Ophiocordyceps* species from NCBI-GenBank databases used in phylogenetic analyses and their associated metadata.

| Species | ID Isolate | GenBank Acession Number | | | Host order | Reference |
|---|---|---|---|---|---|---|
| | | LSU | ITS | TEF | | |
| *Metacordyceps taii* | ARSEF 5714 | AF543787 | - | AF543775 | Lepidoptera | [120] |
| *Metacordyceps taii* | ARSEF 5714 | - | JN049829 | - | Lepidoptera | [53] |
| *Ophiocordyceps arborescens* | NBRC 105891 | AB968414 | AB968398 | AB968572 | Lepidoptera | [121] |
| *Ophiocordyceps arborescens* | NBRC 105890 | AB968415 | AB968399 | AB968573 | Lepidoptera | [121] |
| *Ophiocordyceps australis* | Ophaus 1780 | KC610762 | KP200888 | KC610732 | Hymenoptera | [17] |
| *Ophiocordyceps australis* | Ophaus 774 | KC610764 | n/a | KC610734 | Hymenoptera | [17] |
| *Ophiocordyceps australis* | Ophaus 926 | KC610765 | n/a | KC610735 | Hymenoptera | [17] |
| *Ophiocordyceps australis* | HUATSF 774a | MF416562 | n/a | MF416510 | Hymenoptera | [122] |
| *Ophiocordyceps coccidiicola* | NBRC 100682 | AB968419 | AB968404 | AB968583 | Hemiptera | [121] |
| *Ophiocordyceps coenomyia* | NBRC 108993 | AB968412 | AB968396 | AB968570 | Diptera | [121] |
| *Ophiocordyceps coenomyia* | NBRC 106964 | AB968413 | AB968397 | AB968571 | Diptera | [121] |
| *Ophiocordyceps evansii* | Ophsp 858 | KC610770 | KP200889 | KC610736 | Hymenoptera | [17] |
| *Ophiocordyceps evansii* | Ophsp 912 | KC610771 | KP200890 | KC610737 | Hymenoptera | [17] |
| *Ophiocordyceps gracilis* | EFCC 8572 | EF468811 | - | EF468751 | Lepidoptera | [18] |
| *Ophiocordyceps gracilis* | EFCC 8572 | - | JN049851 | - | Lepidoptera | [53] |
| *Ophiocordyceps heteropoda* | EFCC 10125 | EF468812 | - | EF468752 | Hemiptera | [18] |
| *Ophiocordyceps heteropoda* | EFCC 10125 | - | JN049852 | - | Hemiptera | [53] |
| *Ophiocordyceps irangiensis* | OSC 128577 | DQ518760 | JN049823 | DQ522329 | Hymenoptera | [123] |
| *Ophiocordyceps irangiensis* | OSC 128578 | DQ518770 | JN049833 | DQ522345 | Hymenoptera | [123] |
| *Ophiocordyceps longissima* | NBRC 106965 | AB968420 | AB968406 | AB968584 | Hemiptera | [121] |
| *Ophiocordyceps macroacicularis* | NBRC 100685 | AB968416 | AB968400 | AB968574 | Lepidoptera | [121] |
| *Ophiocordyceps macroacicularis* | NBRC 105888 | AB968417 | AB968401 | AB968575 | Lepidoptera | [121] |
| *Ophiocordyceps myrmecophila* | BCC 82796 | MH028153 | MH028150 | MH028187 | Hymenoptera | [124] |
| *Ophiocordyceps myrmecophila* | BCC 82985 | MH028155 | MH028149 | MH028189 | Hymenoptera | [124] |
| *Ophiocordyceps myrmecophila* | BCC 82258 | MH028159 | MH028147 | MH028190 | Hymenoptera | [124] |
| *Ophiocordyceps myrmecophila* | BCC 82984 | MH028162 | MH028148 | MH028192 | Hymenoptera | [124] |
| *Ophiocordyceps nutans* | NBRC 101749 | JN941429 | - | - | Hemiptera | Okane, I., 2011 (Unpublished) |
| *Ophiocordyceps nutans* | NBRC 101749 | - | AB968408 | AB968589 | Hemiptera | [121] |
| *Ophiocordyceps rhizoidea* | NHJ 12522 | EF468825 | JN049857 | EF468764 | Blattodea (Termite) | [18] |
| *Ophiocordyceps sinensis* | EFCC 7287 | EF468827 | - | EF468767 | Lepidoptera (pupae) | [18] |
| *Ophiocordyceps sinensis* | EFCC 7287 | - | JN049854 | - | Lepidoptera (pupae) | [53] |
| *Ophiocordyceps sobolifera* | NBRC 106967 | AB968422 | AB968409 | AB968590 | Hemiptera | [121] |

| | | | | | | |
|---|---|---|---|---|---|---|
| *Ophiocordyceps stylophora* | OSC 111000 | DQ518766 | - | DQ522337 | Coleoptera | [123] |
| *Ophiocordyceps stylophora* | OSC 111000 | - | JN049828 | - | Coleoptera | [53] |
| *Ophiocordyceps stylophora* | NBRC 100947 | JN941447 | JN943352 | - | Coleoptera (larvae) | [125] |
| *Ophiocordyceps stylophora* | NBRC 100947 | - | - | AB968579 | Coleoptera (larvae) | [121] |
| *Ophiocordyceps thanathonensis* | MFLU 162908 | MF362990 | MF362991 | MF372760 | Hymenoptera | [126] |
| *Ophiocordyceps thanathonensis* | MFLU 162909 | MF850378 | MF850376 | MF872613 | Hymenoptera | [126] |
| *Ophiocordyceps thanathonensis* | MFLU 162910 | MF850377 | MF850375 | MF872614 | Hymenoptera | [126] |
| *Ophiocordyceps tricentri* | NBRC 106968 | AB968423 | AB968410 | AB968593 | Hemiptera | [121] |

**References Supplementary material (Table S1)***

120. Currie, C. R., Wong, B., Stuart, A. E., Schultz, T. R., Rehner, S. A., Mueller, U. G., Soung, G. H., Spatafora, J. W. & Straus, N. A. Ancient tripartite coevolution in the attine ant-microbe symbiosis. Science. 2003, 299, 5605, 386-388.

121. Ban, S., Sakane, T., & Nakagiri, A. Three new species of Ophiocordyceps and overview of anamorph types in the genus and the family Ophiocordyceptaceae. Mycological progress. 2015, 14, 1, 1017.

122. Kepler, R. M., Luangsa-Ard, J. J., Hywel-Jones, N. L., Quandt, C. A., Sung, G. H., Rehner, S. A., Aime, M. C., Henkel, T. W., Sanjuan, T., Zare, R., Chen, M., Li, Z., Rossman, A. Y., Spatafora, J. W. & Shrestha, B. A phylogenetically-based nomenclature for Cordycipitaceae (Hypocreales). *IMA fungus*. 2017. *8*, 2, 335-353.

123. Spatafora, J. W., Sung, G. H., Sung, J. M., Hywel-Jones, N. L., & White Jr, J. F. Phylogenetic evidence for an animal pathogen origin of ergot and the grass endophytes. *Molecular ecology*. 2007, *16*, 8, 1701-1711.

124. Khonsanit, A., Luangsa-ard, J. J., Thanakitpipattana, D., Kobmoo, N., & Piasai, O. Cryptic species within *Ophiocordyceps myrmecophila* complex on formicine ants from Thailand. *Mycological progress*. 2019, *18* (1-2), 147-161.

125. Schoch, C. L., Seifert, K. A., Huhndorf, S., Robert, V., Spouge, J. L., Levesque, C. A., Chen, W. & Fungal Barcoding Consortium. (2012). Nuclear ribosomal internal transcribed spacer (ITS) region as a universal DNA barcode marker for Fungi. *Proceedings of the National Academy of Sciences*, *109*(16), 6241-6246.

126. Xiao, Y. P., Wen, T. C., Hongsanan, S., Sun, J. Z., & Hyde, K. D. (2017). Introducing *Ophiocordyceps thanathonensis*, a new species of entomogenous fungi on ants, and a reference specimen for *O. pseudolloydii*. *Phytotaxa*, *328*(2), 115-126.

*Other citations are listed in the manuscript.

**Table S2:** Proteases and related domains.

| Protein ID | Domain | Clan | Biological process | Molecular function | Cellular component | Feature | Gene |
|---|---|---|---|---|---|---|---|
| NODE_311_length_27694 | Subtilase family | n/a | proteolysis | serine-type endopeptidase activity | cytoplasm | n/a | CDD81_3328 |
| NODE_70_length_116917 | | | | | extracellular space | signal peptide | CDD81_7855 |
| NODE_112_length_83290 | | | | | extracellular space | n/a | CDD81_3718 |
| NODE_133_length_71514 | | | | | cytoplasm | n/a | CDD81_648 |
| NODE_149_length_66206 | Subtilase family; Peptidase inhibitor I9 | n/a; Protease propeptides/inhibitors | proteolysis | serine-type endopeptidase activity | extracellular space | signal peptide | CDD81_2218 |
| NODE_243_length_37519 | | | | | extracellular space | signal peptide | CDD81_253 |
| NODE_67_length_121081 | | | | | cytoplasm | n/a | CDD82_1444 |
| NODE_94_length_88402 | Subtilase family; Peptidase inhibitor I9; Chitin recognition protein | n/a; Protease propeptides/inhibitors; n/a | proteolysis | serine-type endopeptidase activity; chitin binding | nucleus | n/a | CDD81_3985 |
| NODE_70_length_116917 | Subtilase family; Proprotein convertase P-domain | n/a; Galactose-binding domain-like superfamily | proteolysis | serine-type endopeptidase activity | integral component of membrane; plasma membrane | transmembrane alpha helix | CDD81_7829 |
| NODE_235_length_39940 | Peptidase family C54 | Peptidase clan CA | protein transport; autophagy | cysteine-type peptidase activity | nucleus; cytoplasm | n/a | CDD82_1507 |
| NODE_52_length_140965 | Peptidase family M28; PA domain | Peptidase clan MH/MC/MF; LeuD/IlvD-like | n/a | metal ion binding; aminopeptidase activity | extracellular space | signal peptide | CDD81_229 |
| NODE_84_length_99438 | Dipeptidyl peptidase IV (DPP IV) N-terminal region; Prolyl oligopeptidase family | Beta propeller; Alpha/Beta hydrolase fold | proteolysis | serine-type peptidase activity | cytoplasm | n/a | CDD81_6047 |
| NODE_149_length_66206 | Dipeptidyl peptidase IV (DPP IV) N-terminal region; Prolyl oligopeptidase family; Alpha/beta hydrolase family | Beta propeller; Alpha/Beta hydrolase fold | proteolysis | serine-type peptidase activity | extracellular space | signal peptide | CDD81_2227 |
| NODE_42_length_162368 | ERAP1-like C-terminal domain; Peptidase family M1 domain; Peptidase M1 N-terminal domain | Ig-like fold superfamily (E-set); Peptidase clan MA; Baculovirus p35 protein superfamily | n/a | aminopeptidase activity; metallopeptidase activity; zinc ion binding | cytoplasm | n/a | CDD81_2826 |
| NODE_347_length_22686 | | | | | | | CDD81_3652 |
| NODE_103_length_85904 | Metallopeptidase family M24 | n/a | protein initiator methionine removal | metal ion binding; metalloaminopeptidase activity | cytoplasm; extracellular space | n/a | CDD81_6391 |
| NODE_25_length_195320 | Metallopeptidase family M24; Aminopeptidase P, N-terminal domain | n/a; Creatinase/prolidase N-terminal domain superfamily | n/a | aminopeptidase activity; manganese ion binding | mitochondrion | mitochondrial transit peptide | CDD81_1315 |
| NODE_128_length_73625 | | | | | cytoplasm | n/a | CDD81_746 |
| NODE_243_length_37519 | | | | | mitochondrion | n/a | CDD81_258 |

| | | | | | | | |
|---|---|---|---|---|---|---|---|
| NODE_29_length_188585 | Metallopeptidase family M24; zf-MYND-like zinc finger, mRNA-binding; MYND finger | n/a; TRASH superfamily | protein initiator methionine removal | metal ion binding; metalloaminopeptidase activity | cytoplasm | n/a | CDD81_3215 |
| NODE_448_length_10035 | Metallopeptidase family M24; Creatinase/Prolidase N-terminal domain; C-terminal region of peptidase_M24 | n/a; Creatinase/prolidase N-terminal domain superfamily | n/a | metal ion binding; metalloaminopeptidase activity; hydrolase activity | mitochondrion | n/a | CDD81_5383 |
| NODE_73_length_110288 | Aminopeptidase I zinc metalloprotease (M18) | Peptidase clan MH/MC/MF | proteolysis | aminopeptidase activity; metallopeptidase activity; zinc ion binding | cytoplasm | n/a | CDD81_1040 |
| NODE_168_length_57686 | Aminopeptidase I zinc metalloprotease (M18); M42 glutamyl aminopeptidase | Peptidase clan MH/MC/MF | proteolysis | aminopeptidase activity; metallopeptidase activity; zinc ion binding | extracellular space | n/a | CDD81_1337 |
| NODE_147_length_67131 | LysM domain | LysM-like domain | n/a | n/a | nucleus | n/a | CDD81_780 |
| NODE_135_length_70958 | Glycosyl hydrolase family 61 | Ig-like fold superfamily (E-set) | n/a | chitin binding | extracellular space | signal peptide | CDD81_5862 |

**Table S3:** Predicted phospholipase domains for the entomopathogenic fungus *O. australis* CCMB661.

| Protein ID | Domain | Clan | Biological process | Molecular function | Cellular component | Feature | Gene |
|---|---|---|---|---|---|---|---|
| NODE_347_length_22686 | Patatin-like phospholipase; Domain of unknown function | Patatin/FabD/lysophospholipase-like superfamily; n/a | lipid metabolic process | triglyceride lipase activity | organelle membrane | transmembrane alpha helix | CDD82_222 |
| NODE_169_length_56692 | | | | | endomembrane system | transmembrane alpha helix | CDD81_1349 |
| NODE_47_length_145112 | Patatin-like phospholipase; Cyclic nucleotide-binding domain | Patatin/FabD/lysophospholipase-like superfamily; Cupin fold | lipid metabolic process | lysophospholipase activity; phosphatidyl phospholipase B activity | endomembrane system | transmembrane alpha helix | CDD81_4216 |
| NODE_39_length_172552 | Phosphatidylinositol-specific phospholipase C, X domain; Phosphatidylinositol-specific phospholipase C, Y domain | PLC-like phosphodiesterases | lipid metabolic process; signal transduction; intracellular signal transduction | phosphatidylinositol phospholipase C activity | nucleus | n/a | CDD81_4134 |
| NODE_105_length_84742 | | | | | nucleus | n/a | CDD81_5878 |
| NODE_527_length_5624 | | | | | mitochondrion | mitochondrial transit peptide | CDD81_1859 |
| NODE_6_length_286604 | | | | | mitochondrion | n/a | CDD81_3072 |
| NODE_383_length_17045 | Phospholipase D Active site motif | Phospholipase D superfamily | inositol lipid-mediated signaling; lipid catabolic process; phosphatidic acid biosynthetic process | catalytic activity; N-acylphosphatidylethanolamine-specific phospholipase D activity; phospholipase D activity | cytoplasm | n/a | CDD81_7299 |
| NODE_7_length_267218 | Phospholipase D Active site motif; Phospholipase D Active site motif | | | | endomembrane system | transmembrane alpha helix | CDD81_1994 |
| NODE_149_length_66206 | Prokaryotic phospholipase A2 | Phospholipase A2 superfamily | arachidonic acid secretion; phospholipid metabolic process | phospholipase A2 activity | extracellular space | signal peptide | CDD82_2377 |

| | | | | | | | |
|---|---|---|---|---|---|---|---|
| NODE_420_length_13204 | Platelet-activating factor acetylhydrolase, isoform II | Alpha/Beta hydrolase fold | lipid catabolic process | 1-alkyl-2-acetylglycerophosphocholine esterase activity | cytoplasm | n/a | CDD81_6623 |
| NODE_378_length_17628 | Lysophospholipase catalytic domain | Patatin/FabD/lysophospholipase-like superfamily | phospholipid catabolic process | phospholipase activity | mitochondrion | mitochondrial transit peptide | CDD81_7307 |
| NODE_105_length_84742 | | | | | anchored component of plasma membrane | signal peptide, lipidation GPI-anchor | CDD81_6239 |

**Table S4:** Protein tyrosine phosphatase – PTP domains.

| Protein ID | Domain | Clan | Biological process | Molecular function | Cellular component | Feature | Gene | Pfam predicted active site |
|---|---|---|---|---|---|---|---|---|
| **NODE_3_length_322389** | Dual specificity phosphatase, catalytic domain | Phosphatase | dephosphorylation | protein tyrosine/serine/threonine phosphatase activity | cytoplasm | n/a | CDD81_7917 | Similarity to A0A0V0X9Z7 (A0A0V0X9Z7_9BILA) |
| **NODE_1_length_491455** | | | | | mitochondrion | n/a | CDD81_5603 | Similarity to A0A0V0X9Z7 (A0A0V0X9Z7_9BILA) |
| **NODE_111_length_83425** | | | | | mitochondrion | n/a | CDD81_1320 | Similarity to Q93096 (TP4A1_HUMAN) |
| **NODE_486_length_7628** | Dual specificity phosphatase, catalytic domain; Dual specificity protein phosphatase, N-terminal half; Protein-tyrosine phosphatase | Phosphatase | dephosphorylation; protein dephosphorylation; regulation of exit from mitosis | protein tyrosine/serine/threonine phosphatase activity; protein tyrosine phosphatase activity | organelle membrane | transmembrane alpha helix | CDD81_4327 | Similarity to A0A0V0X9Z7 (A0A0V0X9Z7_9BILA) |
| **NODE_88_length_94299** | Dual specificity phosphatase, catalytic domain; Alpha/beta hydrolase family; Alpha/beta hydrolase fold; Serine aminopeptidase, S33; Thioesterase domain | Phosphatase; Alpha/Beta hydrolase fold | dephosphorylation; biosynthetic process | protein tyrosine phosphatase activity; protein tyrosine/serine/threonine phosphatase activity; hydrolase activity, acting on ester bonds | cytoplasm | n/a | CDD81_5141 | Similarity to A1CLZ0 (CCSE_ASPCL); A0A0V0X9Z7 (A0A0V0X9Z7_9BILA) |
| **NODE_371_length_19466** | Low molecular weight phosphotyrosine protein phosphatase | Phosphatase | protein dephosphorylation | acid phosphatase activity; non-membrane spanning protein tyrosine phosphatase activity | extracellular space | n/a | CDD81_6384 | Similarity to P11064 (PPAC_BOVIN) |
| **NODE_2_length_336043** | Myotubularin-like phosphatase domain | Phosphatase | dephosphorylation | protein tyrosine phosphatase activity | cytoplasm | n/a | CDD81_6925 | Similarity to Q9Z2C9 (MTMR7_MOUSE) |
| **NODE_92_length_90100** | Protein-tyrosine phosphatase | Phosphatase | protein dephosphorylation | protein tyrosine phosphatase activity | endomembrane system | transmembrane alpha helix | CDD81_4528 | Similarity to A0A287D394 (A0A287D394_ICTTR) |
| **NODE_143_length_68526** | Rhodanese-like domain | Phosphatase | positive regulation of cell cycle G2/M phase transition | protein tyrosine phosphatase activity | nucleus | n/a | CDD81_4305 | Similarity to A0A0H5C8P2 (A0A0H5C8P2_CYBJN) |
| **NODE_42_length_162368** | Rhodanese-like domain; Protein-tyrosine phosphatase | Phosphatase | protein dephosphorylation | protein tyrosine phosphatase activity | cytoplasm | n/a | CDD81_1533 | Similarity to A0A287D394 (A0A287D394_ICTTR); A0A0Q3SZT0 (A0A0Q3SZT0_AMAAE) |

| | | | | | | | | |
|---|---|---|---|---|---|---|---|---|
| **NODE_19_length_209599** | Tyrosine phosphatase family; Tyrosine phosphatase family | Phosphatase | dephosphorylation | protein tyrosine phosphatase activity | cytoplasm; integral component of membrane | n/a | CDD81_3541 | Similarity to Q4P7L6 (OCA1_USTMA) |
| **NODE_82_length_100911** | Uncharacterized protein | Uncharacterized protein | protein dephosphorylation; dephosphorylation | protein tyrosine phosphatase activity; protein tyrosine/serine/threonine phosphatase activity | mitochondrion | mitochondrial transit peptide | CDD81_2497 | n/a |
